# Metastatic Insulinoma Following Resection of Nonsecreting Pancreatic Islet Cell Tumor: A Case Report and Review of the Literature

**DOI:** 10.1177/2324709612473274

**Published:** 2013-01-01

**Authors:** Anoopa A. Koshy, Ilyssa O. Gordon, Thuong G. Van Ha, Edwin L. Kaplan, Louis H. Philipson

**Affiliations:** 1 University of Chicago Medical Center, Chicago, IL, USA

**Keywords:** nonfunctioning pancreatic endocrine tumor, pancreatic endocrine neoplasm, metastatic insulinoma, recurrent hypoglycemia

## Abstract

A 56-year-old woman presented to our clinic for recurrent hypoglycemia after undergoing resection of an incidentally discovered nonfunctional pancreatic endocrine tumor 6 years ago. She underwent a distal pancreatectomy and splenectomy, after which she developed diabetes and was placed on an insulin pump. Pathology showed a pancreatic endocrine neoplasm with negative islet hormone immunostains. Two years later, computed tomography scan of the abdomen showed multiple liver lesions. Biopsy of a liver lesion showed a well-differentiated neuroendocrine neoplasm, consistent with pancreatic origin. Six years later, she presented to clinic with 1.5 years of recurrent hypoglycemia. Laboratory results showed elevated proinsulin, insulin levels, and c-peptide levels during a hypoglycemic episode. Computed tomography scan of the abdomen redemonstrated multiple liver lesions. Repeated transarterial catheter chemoembolization and microwave thermal ablation controlled hypoglycemia. The unusual features of interest of this case include the transformation of nonfunctioning pancreatic endocrine tumor to a metastatic insulinoma and the occurrence of atrial flutter after octreotide for treatment.

## Introduction

Although hypoglycemia was recognized as early as the 19th century, hyperinsulinism was suspected to be the cause of hypoglycemia in nondiabetic patients.^1^ The first case of a pancreatic islet cell tumor was described by Nicholls in 1902,^2^ but the possibility of an insulinoma was not appreciated until 5 years after the discovery of insulin in 1922. In 1927, Wilder reported the first case of an insulin-secreting tumor.^3,4^ The patient was a surgeon with an 18-month history of severe hypoglycemia; exploratory laparotomy revealed an unresectable insulinoma with hepatic metastases. Unfortunately, the patient died 1 month after surgery.^4^ Wilder and his colleagues tested the idea that the tumor secreted insulin by extracting the patient’s tumor and administering it to rabbits. Their findings demonstrated that tumor extracts produced hypoglycemia and introduced the concept of an insulinoma.^4^ However, it was not until 1929 that Roscoe Graham performed the first surgical resection of an insulinoma, relieving symptoms of hypoglycemia.^3,5^

Insulinomas are the most common pancreatic endocrine tumors, occurring at 4 per 1 million patients per year.^5^ The diagnosis of insulinoma is often delayed due to variability of the clinical presentation and depends on physician awareness.^5^ To our knowledge, there has only been 1 case reported in the literature of a nonfunctional metastatic pancreatic neuroendocrine tumor transforming into an insulin-secreting tumor.^6^ We report a woman with a nonfunctional pancreatic neuroendocrine tumor that transformed into a functional pancreatic endocrine tumor, a metastatic insulinoma.

## Case Report

**Figure 1.**
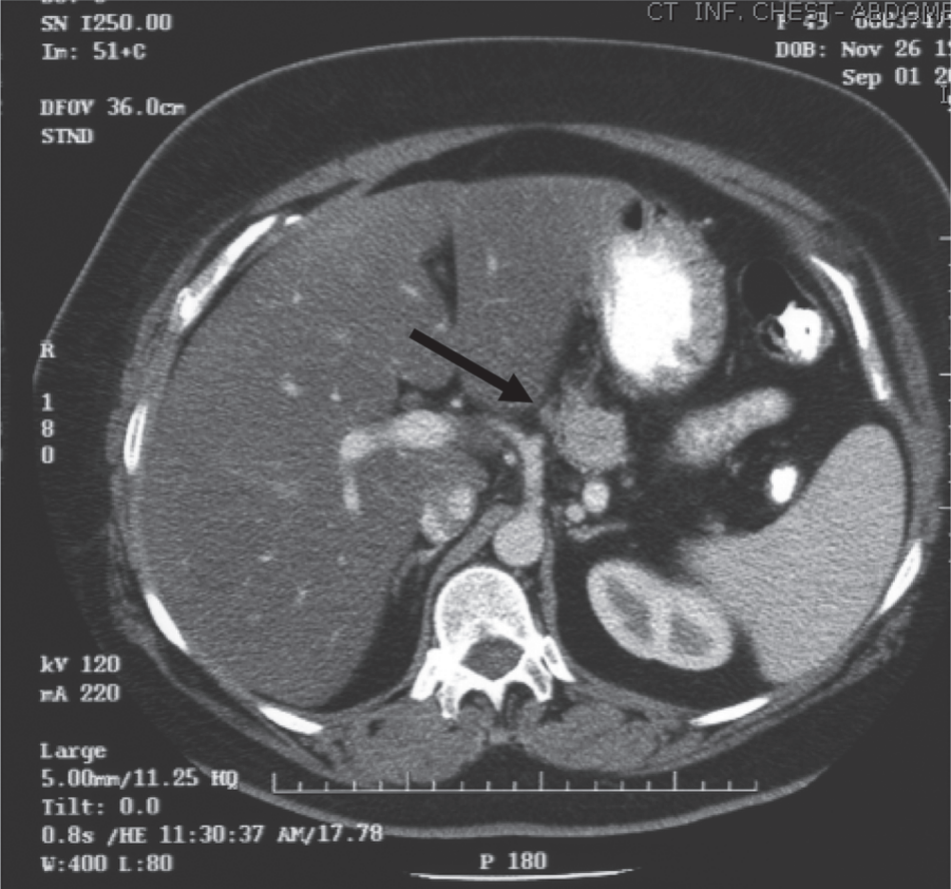
(Date: November 9, 2004) Computed tomography scan of abdomen/pelvis showed an incidental 2.5 × 2.6 cm pancreatic nonsecreting neuroendocrine tumor (arrow)

A 56-year-old woman presented to our clinic for recurrent hypoglycemia. Her past medical history included breast cancer, T1N1M0, diagnosed in 2000 with modified right mastectomy and subsequent chemotherapy with Adriamycin and Cytoxan, radiation therapy, and 5 years of antiestrogen therapy. During surveillance for her breast cancer in 2004, she was found to have an incidental 2.5 × 2.6 cm pancreatic nonsecreting endocrine tumor (Figure 1). She had no symptoms of hypoglycemia at the time, but she did have an elevated glucose level and was taking 35 units of glargine at bedtime and glyburide. Her fasting glucose value off insulin and glyburide (November 10, 2004) was 205 mg/dL, with an insulin level of 36 uIU/mL, a glucagon value of 66 (normal <60), a gastrin value of 83 pg/mL (<100 pg/mL), and a pancreatic polypeptide value of 205 pg/mL (70-430 pg/mL). Her serum calcium level was 9.4 mg/dL.

**Figure 2.**
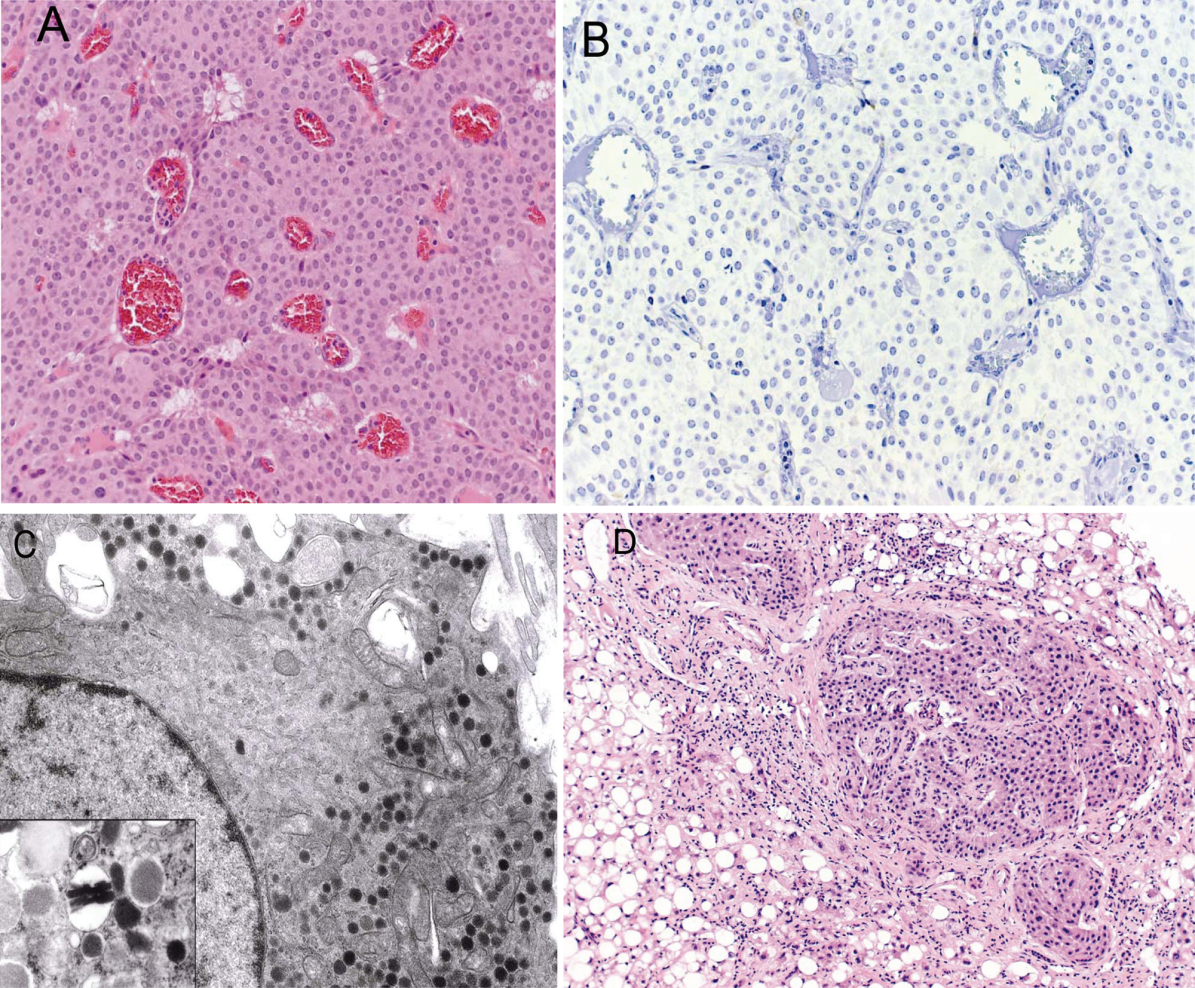
(A) Pancreatic tumor histology. Representative hematoxylin and eosin–stained section of resected pancreatic mass showed a well-differentiated endocrine tumor (original magnification, 20×). (B) Immunohistochemical staining for insulin was negative (original magnification, 20×). (C) Electron micrograph of pancreatic tumor. The cytoplasm contained round neuroendocrine granules with dense cores; none of the granules had the typical crystalloid structure of insulin granules (original magnification, 6000×); inset: example of crystalloid insulin granules from a normal islet (original magnification, 20 000×). (Images courtesy Elizabeth Sengupta, MD, and Jerome Taxy, MD, The University of Chicago Medical Center.) (D) Biopsy of liver lesion showed a well-differentiated endocrine neoplasm (hematoxylin and eosin stain; original magnification, 10×)

She underwent an exploratory laparotomy on January 26, 2005, and a mass was found in the body of the pancreas. No tumor was palpable in the liver or elsewhere in the abdominal cavity. A distal pancreatectomy with splenectomy was performed. The pathologic specimen showed a 2.1-cm neuroendocrine tumor in the body of the pancreas (Figure 2A). The resection margin and other margins were negative for the tumor without capsular or vascular invasion and 0/13 lymph nodes were tumor positive. Immunohistologic stains of the tumor for insulin, glucagon, and somatostatin were negative (Figure 2B). Electron microscopy demonstrated cells with nonspecific granules with no evidence of insulin-type granules (Figure 2C). Postoperatively, her diabetes worsened and she was placed on an insulin pump.

In 2007, a computed tomography (CT) scan of the abdomen and pelvis showed multiple liver lesions. Biopsy of the liver lesion showed a metastatic well-differentiated neuroendocrine neoplasm (Figure 2D). She was diagnosed with stage IV disease and managed conservatively. In 2009, her insulin requirements declined. Eventually she stopped the pump as she required no insulin and was off insulin for 8 months prior to presentation to our clinic in April 2011. She noted increasing episodes of hypoglycemia over the past 1.5 years, several times a day, most frequently after meals. Her symptoms of diaphoresis and tachycardia prior to hypoglycemia improved with eating carbohydrates. She also reported a weight gain of 60 lbs in 5 months from overeating to compensate for the low blood sugars.

Her past medical history was significant for breast cancer, coronary artery disease status post 3 stents, hypertension, hyperlipidemia, obesity, and arthritis. Her medications included clopidogrel, metoprolol succinate, zolpidem, hydrochlorothiazide/triamterene, and atorvastatin. She was married with 4 children, including 1 son who died of cardiomyopathy. No family history of pancreatic tumors or diabetes was noted.

During a period of a few months before her initial clinic visit, she underwent several tragedies in her personal life, including loss of her son at age 35, death of her father from prostate cancer, and the sudden death of her father-in-law. Her doctors noted this and attributed her recent symptoms to the stressors in her life. Her primary care doctor prescribed diazoxide 200 mg twice a day with modest improvement in her glucose levels, so they rarely went below 70 mg/dL. She did not tolerate the taste of diazoxide and stopped it a month before her clinic visit.

**Figure 3.**
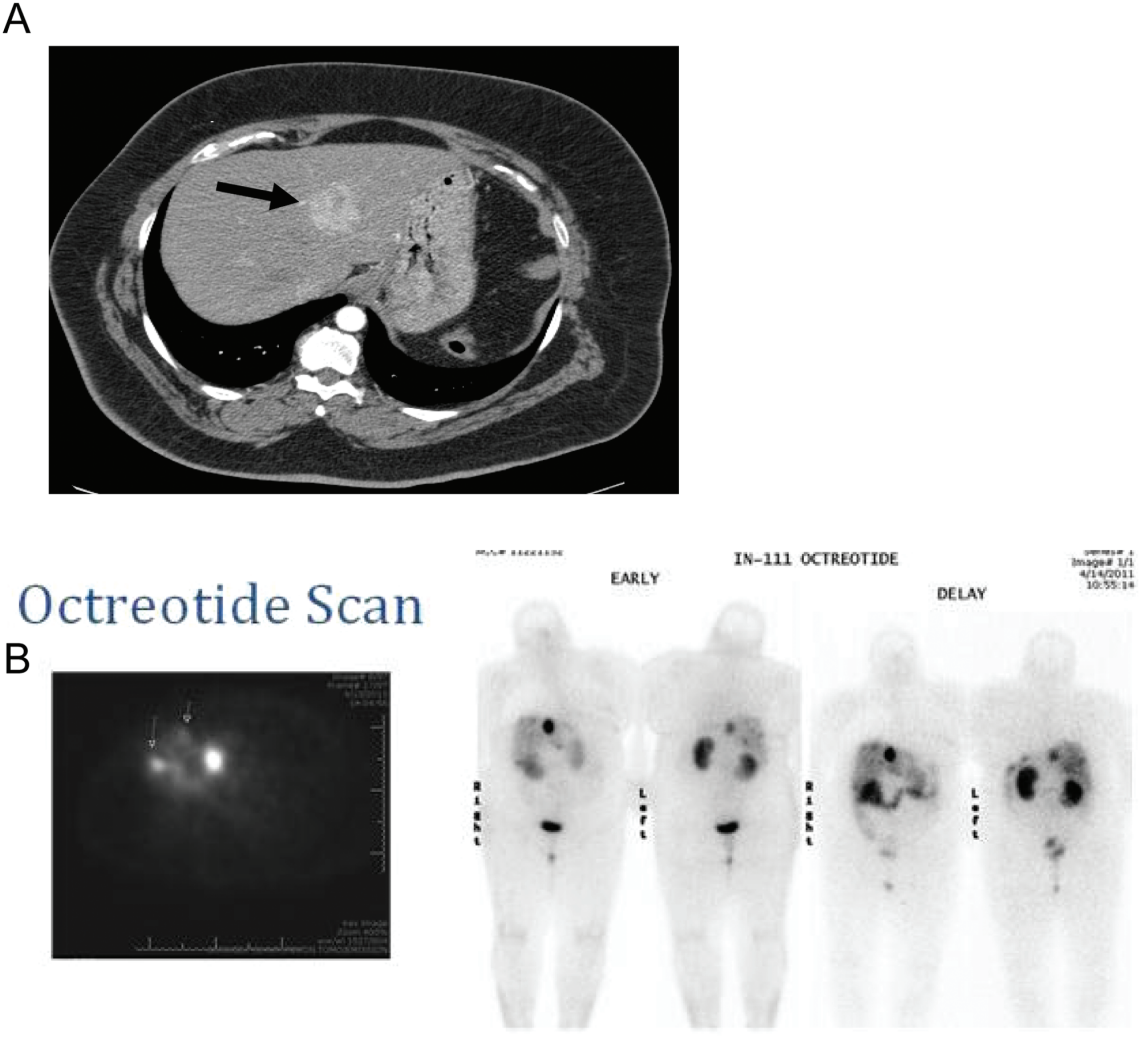
(A) Contrast-enhanced CT of the abdomen/pelvis (date: April 13, 2011) showed a dominant left lobe mass (arrow). Smaller liver lesions were also present (not shown). (B) Octreotide scan (date: April 14, 2011) showed a focus of intense uptake in the right lobe of the liver consistent with neuroendocrine metastasis, and numerous additional smaller foci of increased uptake are noted in the liver consistent with metastatic disease (small arrows)

She continued to have low blood sugars in the 30s to 40s mg/dL range. Her blood glucose in clinic was 53 mg/dL and with symptoms of diaphoresis, tachypnea, and tachycardia (92 bpm). A critical blood sample was drawn. Her blood glucose was 64 mg/dL with a c-peptide of 6.76 pmol/mL (normal range 0.3-2.35). Her insulin level was elevated to 578 uIU/mL with a proinsulin of 4300 pmol/L (normal range 3-20) and glucagon of 53 pg/mL (normal <80). Her serum cortisol at 3:43 pm was 18.2 µg/dL. Her vasoactive intestinal peptide was 28 pg/mL (normal <75) with a chromogranin A of 2040 ng/mL (normal <225), free thyroxine of 0.92 ng/dL (normal range 0.9-1.7), thyroid-stimulating hormone of 2.97 µU/mL (normal range 0.3-4), and HbA1c of 4.9%. She underwent a CT scan of her abdomen and pelvis on April 13, 2011, which showed multiple hepatic metastases, the largest of which increased in size since the previous exam (see Figure 3A).

She underwent an octreotide scan that showed a focus of intense uptake in the right lobe of the liver consistent with neuroendocrine metastasis and numerous additional smaller foci of increased uptake in the liver consistent with metastatic disease (see Figure 3B). She was prescribed prednisone 40 mg daily, encouraged to use corn starch mixed with water at bedtime, and given a prescription for glucagon emergency kit for extreme hypoglycemia.

**Figure 4.**
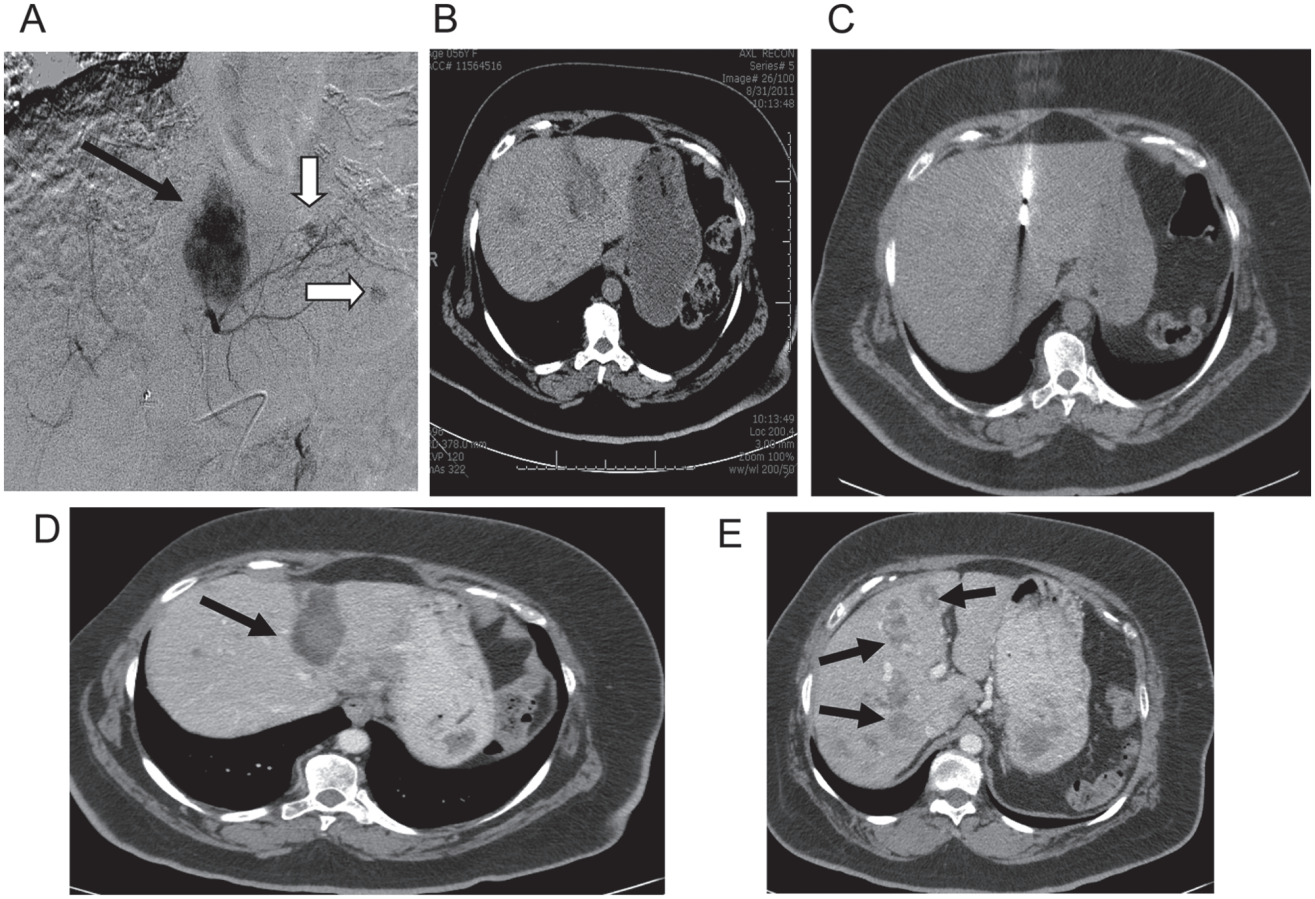
(A) (Date: April 25, 2011) Angiogram of the left hepatic artery showed the tumoral blush (black arrow) corresponding to the large mass on CT. Note other smaller tumoral blushes (white arrows). (B) (Date: August 31, 2011) CT of abdomen and pelvis showed residual enhancement in the large hepatic metastases near the dome of the liver. In addition, there are numerous other focal metastatic lesions visualized in both lobes of the liver. (C) (Date: September 9, 2011) CT image performed during thermoablation showed the antenna in the location of the mass. Ablation was performed because there was inadequate clinical response to chemoembolization alone. (D) (Date: January 5, 2012) Follow-up CT of abdomen/pelvis showed good control of the dominant lesion (arrow) without enhancement (arrow). (E) Same CT as in “D” at different level of the liver showed other metastatic hepatic lesions (arrows)

On April 21, she was admitted from clinic with recurrent hypoglycemia. She was started on prednisone 20 mg 3 times a day and continuous infusion of D5½ normal saline. She underwent transcatheter arterial chemoembolization (TACE) of left hepatic lobe for symptom control (Figure 4A). The next day, D5½ normal saline was stopped, and prednisone continued. Two days after TACE, her proinsulin level decreased from 2300 on 4/8 to 263 pmol/L. Her fasting blood glucose was 95 mg/dL with a c-peptide of 2.03 pmol/mL at the time (see Table 1).

**Table 1.** Biochemical Data

	April 8, 2011	April 27, 2011	April 30, 2011	May 23, 2011	August 22, 2011	September 8, 2011	December 9, 2011	April 20, 2012
Treatment	TACE—April 25, 2011	TACE—May 2, 2011				TACE—September 8, 2011	TACE—January 25, 2012	TACE—April 30, 2012
		Ablation—May 3, 2012				Ablation—September 9, 2012	Ablation—January 26, 2012	Ablation—May 1, 2012
Medications	Prednisone	Octreotide, glargine		70/30 Insulin	Insulin pump		Insulin pump	Insulin pump
Proinsulin (3-20 pmol/L)	4300	263	170	220	470		380	780
c-Peptide (0.3-2.35 pmol/mL)	6.76	2.03		1.1	1.47		1.42	2.09
Insulin (uIU/mL)	578			192				95
Glucose (mg/dL)	64	95	109		106		121	
Chromogranin A (<225 ng/mL)	2040		1780	360	425		815	

Abbreviation: TACE, transcatheter arterial chemoembolization.

Due to persistent hypoglycemia, she was given octreotide at 150 µg subcutaneously, which caused marked hyperglycemia with blood glucoses above 500 mg/dL. The dose of octreotide was reduced to 50 µg, but still caused hyperglycemia. The patient refused further doses but then developed atrial flutter. With diltiazem and metoprolol she converted back to normal sinus rhythm. On the next day, she became hypoglycemic again and underwent a second round of TACE of left and right hepatic lobes with microwave thermal ablation. She was monitored overnight and her blood sugars improved. She became hyperglycemic the next day; the prednisone was stopped, and she was started on a low dose of glargine. Her proinsulin level after her second chemoembolization procedure was 220 pmol/L and insulin was 192 uIU/mL. She was discharged home.

She returned to Endocrinology clinic 3 days later, was noted to be tachycardic, and EKG obtained again showed atrial flutter. She was administered diltiazem and then sotalol and converted to normal sinus rhythm. Due to hyperglycemia, her insulin was changed to insulin 70/30, 10 units twice a day.

She denied any hypoglycemic episodes at a follow-up visit to the Endocrine clinic the following month, but she had been using less insulin. CT scan of her abdomen and pelvis showed residual enhancement in the large hepatic metastases near the dome of the liver with numerous other focal metastatic lesions visualized in both lobes of the liver (Figure 4B).

She underwent repeat TACE and microwave thermal ablation on the following day (Figure 4C). She was hyperglycemic after the procedure and discharged home with low-dose insulin aspart adjustment instructions. She was subsequently placed back on the insulin pump.

A repeat CT scan of her abdomen and pelvis several months later showed an increase in the size and number of hypodense and enhancing components lesions in the liver. Her HbA1c was 8.9% without hypoglycemic events. Her c-peptide was 1.42 pmol/mL, but her proinsulin level was elevated at 380 pmol/L. A repeat TACE with microwave thermal ablation was performed. Follow-up CT (January 2012) showed good control of the dominant liver lesion without enhancement (Figure 4D); however, it also showed other metastatic hepatic lesions present (Figure 4E).

Most recently, CT scan of her abdomen and pelvis on April 16 showed a slight decrease in the largest lesion in the liver with smaller nodular components. She was not experiencing recurrent hypoglycemia, and did not need exogenous insulin for the previous 17 days. Her proinsulin level rose to 780 uIU/mL with an insulin level of 95 uIU/mL and a c-peptide of 2.09 pmol/L. She underwent another repeat TACE with microwave thermal ablation, after which she has remained symptom-free until hypoglycemia resulted in another subsequent round of TACE and thermal ablation.

## Discussion

Pancreatic endocrine tumors are usually well differentiated and classified as functional or nonfunctional based on their ability to secrete hormones.^7^ Functional tumors secrete hormones including insulin, glucagon, vasoactive intestinal peptide, somatostatin, and rarely neurotensin, growth hormone–releasing factor, and adrenocorticotropic hormone. Nonfunctional tumors comprise approximately 30% of pancreatic endocrine tumors, and the majority of these will have metastases at presentation.^5,8^ Of patients with insulinomas, approximately 80% have single, usually small (<1.5 cm) tumors.^9^ Only 10% have malignant tumors and the remaining 10% have multiple benign lesions.^10^ Unfortunately, other than insulinomas, most pancreatic endocrine tumors are frequently diagnosed at a late stage, with approximately 65% of patients presenting with unresectable or metastatic disease. These patients have a poor prognosis with the median survival time of 24 months.^11^

Our patient had a nonfunctioning pancreatic endocrine tumor at diagnosis and developed multiple hepatic metastases, which later became functional for insulin secretion approximately 6 years after the initial diagnosis. The nonsecreting metastatic tumor in this patient transformed into a malignant insulinoma with dramatic insulin and proinsulin secretion. The metastatic lesions of an insulinoma generally secrete excessive insulin, identical to the primary tumor. The significance of the neuroendocrine granules seen on electron microscopy of the pancreatic tumor in this case are not clear, since they can be present in normal patients’ islet cells, but the contents of these granules, if secreted, did not seem to be clinically significant. One possibility is that the original tumor had multiple components, including a nonfunctional tumor and an undetectable insulinoma component of malignant potential that had already metastasized to the liver at the time of the distal pancreatectomy. This is less likely since the hepatic metastases were present for at least 3 years without the patient having symptoms of a functioning tumor. The insulinoma component may have been growing slowly, so not enough insulin was being secreted to cause initial symptoms. The tumor cells could have redifferentiated, developing the ability to secrete insulin, not present at time of resection. The tumor cells gained the ability to express the proinsulin gene along with components of the prohormone convertases specific for insulin production. This is further supported by the fact that In fetal development, nonhormonal components such as chromogranins appear in the pancreatic endocrine cells earlier than specific islet hormones.^12^

Many therapeutic options have been used to treat hypoglycemia in insulinoma patients with unresectable metastases. Octreotide, used successfully in the treatment of insulinomas, in this patient was associated with development of atrial flutter. Alberts and Falkson reported a 52-year-old man with metastatic insulinoma who responded rapidly to octreotide and remained responsive 4 months later.^13^ The response rate varies among patients and may be correlated with the degree of expression of somatostatin receptor subtypes 2 and 3. The biological half-life of octreotide is about 100 minutes, so 2 to 3 doses per day are needed to prevent hypoglycemia in insulinoma patients. Interestingly, our patient developed atrial flutter not long after the administration of octreotide subcutaneously. Cardiac dysrhythmias have a 10% occurrence rate with octreotide. Bradycardia, arrhythmia, conduction abnormalities, and other EKG changes may also occur. The relationship of these events to octreotide is not clearly established because many of these patients have underlying cardiac disease, so caution is recommended when administering octreotide to at-risk patients.^14^ Our patient did have underlying cardiac disease, and it is possible she was at high risk and predisposed to the development of atrial flutter after octreotide.

Our patient also underwent TACE and microwave thermal ablation multiple times with relapse of her hypoglycemia a few months after each procedure. Additional options are limited. Everolimus, an oral inhibitor of mammalian target of rapamycin (mTOR), has shown antitumor activity in patients with advanced pancreatic neuroendocrine tumors in a prospective, randomized, phase 3 study.^15^ Everolimus, compared with placebo, significantly prolonged progression-free survival among patients with progressive advanced pancreatic neuroendocrine tumors (11 months compared with 4.6 months with placebo) and was associated with a low rate of severe adverse events.^15^ Fiebrich and colleagues also administered everolimus to 4 insulinoma patients and showed that it normalized plasma glucose levels in metastatic insulinoma within 14 days, coinciding with a lower glucose uptake in tumor and muscles and declining proinsulin levels.^16^ Given the positive response seen in these patients described in the literature, such patients should be considered for everolimus protocol for intractable hypoglycemia. Radioembolization using yttrium-90-labeled resin or glass microspheres also has been shown to offer effective disease control and possible improved quality of life and can be considered as an option for both functional and nonfunctional pancreatic endocrine tumors.^17^ Liver transplant is another option that may be considered if tumor is confined to the liver.

In summary, we report an unusual case of a nonfunctional pancreatic neuroendocrine tumor that transformed into a functional tumor, a metastatic insulinoma. Transcatheter arterial chemoembolization with microwave thermal ablation was used to help with symptom control. Octreotide, used for treatment of hypoglycemia, may have resulted in the occurrence of atrial flutter.
